# DNA Damage Response Network and Intracellular Redox Status in the Clinical Outcome of Patients with Lung Cancer

**DOI:** 10.3390/cancers16244218

**Published:** 2024-12-18

**Authors:** Dimitra Mavroeidi, Anastasia Georganta, Dimitra T. Stefanou, Christina Papanikolaou, Konstantinos N. Syrigos, Vassilis L. Souliotis

**Affiliations:** 1 Institute of Chemical Biology, National Hellenic Research Foundation, 11635 Athens, Greece; dmavro@eie.gr (D.M.); chrpapa@eie.gr (C.P.); 2 Third Department of Medicine, Sotiria General Hospital for Chest Diseases, National and Kapodistrian University of Athens, 11527 Athens, Greece; anastgew@med.uoa.gr (A.G.); ksyrigos@med.uoa.gr (K.N.S.); 3 First Department of Internal Medicine, Laikon General Hospital, School of Medicine, National and Kapodistrian University of Athens, 11527 Athens, Greece; dimitroulastef@hotmail.com

**Keywords:** DNA damage response (DDR), lung cancer, PBMCs, platinum-based chemotherapy, clinical response, redox status, oxidative stress, apurinic/apyrimidinic (AP) sites, nucleotide excision repair (NER), interstrand cross-link repair (ICL/R)

## Abstract

**Background/Objectives**: DNA damage response (DDR) is a network of molecular pathways associated with the pathogenesis and progression of several diseases, as well as the outcome of chemotherapy. Moreover, the intracellular redox status is essential for maintaining cell viability and controlling cellular signaling. Herein, we analyzed DDR signals and redox status in peripheral blood mononuclear cells (PBMCs) from patients with lung cancer with different response rates to platinum-based chemotherapy. **Methods**: Several DDR-associated signals and redox status, expressed as the GSH/GSSG ratio, were measured in two lung cancer cell lines (A549, H1299), two normal fibroblast cell lines (WS1, 1BR3hT), and PBMCs from 20 healthy controls and 32 patients with lung cancer at baseline (17 responders and 15 non-responders to subsequent platinum-based chemotherapy). **Results**: Higher levels of endogenous/baseline DNA damage, decreased GSH/GSSG ratios, and augmented apurinic/apyrimidinic sites, as well as lower nucleotide excision repair (NER) and increased interstrand cross-links (ICLs) repair efficiencies, were observed in lung cancer cell lines compared with normal ones (all *p* < 0.05). Moreover, PBMCs from patients with lung cancer showed reduced GSH/GSSG ratios, augmented apurinic/apyrimidinic sites, decreased NER and ICL repair capacities, and lower apoptosis rates, compared with healthy controls (all *p* < 0.001). Interestingly, PBMCs from patients who are responders are characterized by reduced GSH/GSSG ratios, augmented apurinic/apyrimidinic sites, decreased NER and ICL repair capacities, and higher apoptosis rates compared with patients who are non-responders (all *p* < 0.01). **Conclusions**: Together, DDR-associated parameters and redox status measured in PBMCs from patients with lung cancer at baseline are associated with the therapeutic benefit of platinum-based chemotherapy.

## Simple Summary

DNA damage response (DDR) is a multi-factor network that is responsible for the removal of DNA lesions, thus enabling cells to function normally. An imbalance between the generation of reactive oxygen species and their removal by defense mechanisms is known as oxidative stress. Previous studies demonstrated that deregulation of the DDR network and redox imbalance are implicated in the onset and progression of several diseases, including cancer, as well as in the outcome of chemotherapy. In this study, we found that DDR-associated parameters and the intracellular redox status display significant differences among patients with lung cancer at baseline and correlate with the clinical responses to subsequent platinum-based therapy. The exploitation of these results might lead to the identification of new therapeutic targets, the design of effective and sensitive biomarkers, and the development of new therapeutic regimens for the treatment of this devastating malignancy.

## 1. Introduction

Lung cancer remains one of the most-diagnosed malignant diseases over decades, characterized by high mortality rates, with over 2 million cases per year rising worldwide [1]. Based on the histology of the cancer cells, lung cancer can be categorized into two types: small cell lung cancer (SCLC), which comprises about 15% of lung cancers, and non-small cell lung cancer (NSCLC), which accounts for 85% of all cases and can be further subdivided into three histological types: adenocarcinoma, large cell carcinoma, and squamous cell carcinoma [2]. Despite considerable progress in understanding, diagnosing, and treating the disease, further advances seem obligatory. One major challenge appears to be the identification of accurate predictive biomarkers that can be utilized in the clinic to improve treatment design [3].

Lung cancer has early been related to cigarette smoking, and many carcinogenic compounds have been identified in tobacco ever since [4]. These substances induce DNA damage, contributing to genomic instability associated with the high mutational burden of lung cancer cells. Nevertheless, a significant percentage of patients who develop lung cancer have not ever used tobacco, implying further DNA-damaging factors, such as environmental pollution, radiation, exposure to industrial hazardous agents, etc., pose as risk factors, interplaying with genetic predisposition [5,6].

The human genome is constantly exposed to multiple exogenous (genotoxic chemicals, UV light, ionizing radiation, etc.) and endogenous DNA-damaging factors (e.g., oxidation, alkylation, hydrolysis, and mismatch of DNA bases) [7,8,9]. Genotoxic stress may also emerge from several cellular processes, such as replication and transcription [10,11]. Specifically, the generation and response to reactive oxygen species (ROS) remain among the most well-studied genotoxic mechanisms, as cancer cells are frequently characterized by impaired regulation of ROS. Several pathways are involved in ROS regulation, as their function is critical for cell signaling and metabolism. Disrupted ROS levels lead to pathological outcomes and disease development [12,13], while redox status has been highlighted as critical for both cancer progression and chemotherapy response [14]. Particularly in lung cancer, evidence suggests that oxidative stress is critical for the onset and progression of the disease, as lungs are more susceptible due to their exposure to oxygen and blood circulation [15]. Moreover, ROS are capable of directly inducing DNA lesions, including oxidized purines and pyrimidines, single-strand breaks (SSBs), double-strand breaks (DSBs), and abasic (AP; apurinic/apyrimidinic) sites [16,17,18]. Specifically, AP-sites are common DNA lesions that may occur both spontaneously, due to oxidative stress, and as intermediates of DNA repair pathways, such as base excision repair (BER) [19]. Repair of AP-sites includes their cleavage and SSB formation that may result in DSBs during DNA replication. As such, AP-sites levels have been suggested as a possible biomarker for oxidative stress and BER capacity, while it has been proposed that it could even predict survival in patients with resected NSCLC [19]. Moreover, a key factor of BER, the apurinic/apyrimidinic endonuclease 1 (APE1), has been underlined as a therapeutic target in NSCLC, as its inhibition resulted in excessive DNA damage and augmented tumor cell death in vitro and in vivo [20].

Interestingly, genotoxic drugs like platinum-based compounds have been reported to induce oxidative stress-related cytotoxic effects, either by directly generating ROS or by blocking the antioxidant system [21,22,23]. As a result, redox status appears to be crucial for response to chemotherapy-based treatment [14,24,25,26]. Glutathione, a key antioxidant factor, has been found to react with cisplatin and regulate resistance to this drug [26,27,28,29,30]. GSH, the reduced form of glutathione, binds and deactivates cisplatin molecules, thus preventing them from reaching the DNA and forming adducts [28,29]. In parallel, GSH reacts with the cisplatin-induced ROS, interfering with ROS-mediated cytotoxicity [29]. In particular, cisplatin cytotoxicity depends on the glutathione levels and the expression of the nuclear factor erythroid 2-related factor 2 (NRF2), which controls the transcription of glutathione components in lung cancer [29] and other tumor cells [31].

To confront the above challenges and ensure genomic stability, cells have developed a complex system of molecules and pathways, commonly known as the DNA damage response (DDR) network, including sensors of the lesion sites, cell cycle kinases, signaling cascades and effector proteins that maintain genomic integrity [32,33]. Disruption of the function of DDR network contributes to genomic instability and is involved in tumorigenesis. Furthermore, recent data have shown that DDR strongly impacts the immune system suggesting crucial therapeutic implications [34,35]. Specifically, defects in repair mechanisms may lead to the accumulation of cytosolic DNA resulting in stimulation of innate immune response and/or genomic mutations. These effects increase tumor mutational burden and levels of MHC-presented neoantigens, thus potentiating anti-tumor immune response [36,37,38].

Lung cancer is currently commonly treated with chemotherapy, molecular-targeted therapy, immunotherapy, radiation therapy and surgery [1,2]. The standard of care for most advanced NSCLC-patients includes platinum-based chemotherapy [39], thanks to its cytotoxicity. These drugs function by inducing the formation of DNA monoadducts that are almost exclusively repaired by nucleotide excision repair (NER), and interstrand cross-links (ICLs), which are repaired by NER, translesion synthesis, Fanconi anemia pathway, homologous recombination (HR), and nonhomologous end-joining (NHEJ) [18].

In this study, we tested the hypothesis that redox status and DDR-related parameters of patients with lung cancer at baseline correlate with therapeutic benefit from subsequent platinum-based treatment. Towards this, we evaluated GSH/GSSG ratio (a reliable estimation of cellular redox status), apurinic/apyrimidinic sites, and several DDR parameters, including the endogenous/baseline DNA damage, the efficiencies of critical DNA repair mechanisms and the apoptosis rates in normal and lung cancer cell lines, as well as in peripheral blood mononuclear cells (PBMCs) from healthy controls and patients with lung cancer at baseline.

## 2. Materials and Methods

### 2.1. Patients

A total of 32 patients with lung cancer were included in this study: seventeen (*n* = 17) patients with partial response to therapy (PR; 3 females/14 males; median age, 66 years; range, 49–82), five (*n* = 5) with stable disease (SD; 2 females/3 males; median age, 68 years; range, 65–76), and ten (*n* = 10) with progressive disease (PD; 3 females/7 males; median age, 68.5 years; range, 62–81) (Table 1). Twenty (*n* = 20) healthy individuals were also included as controls (HC; 8 females/12 males; median age 61.4 years; range, 41–82). PBMCs were isolated from freshly drawn peripheral blood and purified using the standard Ficoll gradient centrifugation. Briefly, 10 mL of whole blood were diluted 1:1 in RPMI-1640 medium, carefully layered over 15 mL of Ficoll reagent (Ficoll-Paque PLUS, Sigma-Aldrich, St. Louis, MI, USA, #GE17-1440-03), and centrifuged at 400× *g* for 20 min at 20 °C, without brake or acceleration. After centrifugation, the semi-white layer containing PBMCs was collected using a syringe. The collected cells were transferred to 10 mL of RPMI-1640 medium and washed twice via centrifugation at 400× *g* for 20 min at 20 °C, this time with normal brake and acceleration. Cells were resuspended in freezing medium (90% fetal bovine serum and 10% dimethyl sulfoxide) and stored at −80 °C until further processing. The study was approved by the Institutional Review Board of Soteria Hospital (No. 15627/11.6.2020 and No. 25950/10.10.2022), and all subjects provided informed consent. The study was conducted according to the Declaration of Helsinki.

### 2.2. Cell Lines

Human 1BR3hT cells (immortalized normal skin fibroblasts) and H1299 cells (epithelial-like non-small-cell lung carcinoma) were maintained in Dulbecco’s modified Eagle’s medium (DMEM), 10% fetal bovine serum (FBS), and 1% penicillin/streptomycin (Pen/Strep). Human A549 cells (non-small-cell lung carcinoma) were maintained in DMEM/Ham’s F12 (1:1) medium, 10% FBS, and 1% Pen/Strep. Human WS1 cells (normal skin fibroblasts) were maintained in DMEM, 1% non-essential amino acids, 10% FBS, and 1% Pen/Strep.

### 2.3. Measurement of Nucleotide Excision Repair (NER)—Alkaline Comet Assay

DNA damage was assessed using the alkaline comet assay. Cells suspended in phosphate-buffered saline (PBS; pH 7.4) were mixed with 1% low-melting-point agarose and loaded onto glass slides. The slides were allowed to solidify at 4 °C for 30 min, incubated in an alkaline lysis buffer (2.5 M NaCl, 0.1 M EDTA, 0.01 M Tris; pH 10, 1% Triton X-100) for 2 h at 4 °C, and electrophoresed at 25 V, 225 mA for 30 min. The slides were stained with SYBR™ Gold nucleic acid gel stain (Thermo Fisher Scientific, Waltham, MA, USA, #S11494) and imaged under UV light using a 10× microscopy lens. Olive tail moment (OTM) was analyzed using ImageJ Analysis/OpenComet v1.3.1 (https://cometbio.org/). For each sample, 2 gels were scored, and the average OTM value of 150 cells was calculated.

### 2.4. Measurement of Gene-Specific Repair of the Interstrand Cross-Links

Cell lines or PBMCs were treated with cisplatin (25 μg/mL and 5 μg/mL, respectively) for 3 h at 37 °C in the appropriate culture medium, incubated in drug-free medium for 0–24 h at 37 °C, harvested, and stored in freezing medium at −80 °C. The gene-specific repair of the ICLs was measured using Southern blot analysis, as described previously [40]. For the analysis of N-ras alkylation, after isolation, genomic DNA was digested using the restriction enzyme EcoRI, and DNA samples were denatured prior to gel electrophoresis. That is, DNA was incubated at 37 °C for 15 min in 50 mM NaOH, denaturation was stopped on ice, and DNA samples were mixed with sample loading buffer (final concentration: 0.2% Ficoll, 0.1 mM EDTA, 0.01% bromocresol green). Electrophoresis was performed for 16 h at 30 V in 0.6% agarose gel, using buffer containing 40 mM Tris-acetate and 2 mM EDTA. Hybridizations were performed as previously described [41]. The percentage cross-linking, which is the density of the cross-linked DNA band as a fraction of both single-stranded and cross-linked band densities, was used to calculate the frequency of ICLs. The average number of ICLs per restriction fragment examined was calculated using the formula of the Poisson distribution: (cross-links/fragment) = −loge(fraction of fragment free of cross-links).

### 2.5. GSH/GSSG Ratio and Abasic Sites

The GSH/GSSG ratio was determined using a luminescence-based assay that measures total glutathione (GSH+GSSG), oxidized glutathione (GSSG), and the GSH/GSSG ratio, following the manufacturer’s instructions (GSH/GSSG-Glo Assay, Promega, Madison, WI, USA, #V6612). Abasic sites were analyzed with the OxiSelect Oxidative DNA Damage Quantitation Kit (Cell Biolabs, San Diego, CA, USA; #STA-324), also in accordance with the manufacturer’s protocol.

### 2.6. Apoptosis Rates

PBMCs were exposed to varying doses of cisplatin (0–150 μg/mL) for 3 h at 37 °C in complete RPMI-1640 medium, followed by a 24 h incubation in cisplatin-free medium. Apoptosis rates were measured using the Cell Death Detection ELISA PLUS kit (Roche Diagnostics Corp., #11.774.425.001, Mannheim, Germany) according to the manufacturer’s instructions.

### 2.7. Western Blot Analysis

Cell lysates were prepared using the RIPA Lysis Buffer System (Santa Cruz Biotechnology, Dallas, TX, USA, #sc-24948). Protein electrophoresis was carried out on 4–20% FastGene PAGE Gels (Nippon Genetics, Tokyo, Japan, #PG-S420) with MOPS buffer (Nippon Genetics, #PG-MOPS10). Proteins were transferred to nitrocellulose membranes (GE Healthcare, Chicago, IL, USA, Amersham Protran 0.45 μm, #10600002) and incubated overnight at 4 °C with primary antibodies (Cell Signaling Technology, Danvers, MA, USA; γH2AX, #80312; β-tubulin, #15115L; β-actin, #3700). Membranes were incubated with horseradish peroxidase (HRP)-conjugated secondary antibodies (Cell Signaling Technology; anti-mouse IgG: HRP, #7076S; anti-rabbit IgG: HRP, #7074S), the antibody complexes were visualized using the Pierce™ ECL Western Blotting Substrate (Thermo Scientific, #32106) and imaged using the BioRad Gel Doc XR Imaging System.

### 2.8. Statistical Analysis

An unpaired *t* test with Welch’s correction was applied for *p*-value determination. The results were of statistical significance when *p* < 0.05. All statistical analyses and graph design were carried out with GraphPad Prism 8.0.1. The mean ± SD was used to present the data.

## 3. Results

### 3.1. DDR-Associated Parameters in Lung Cancer Cell Lines

**Figure 1:**
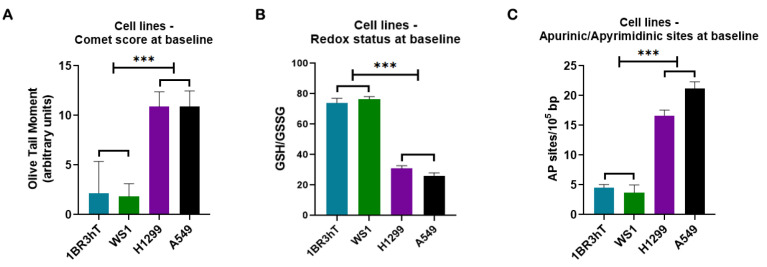
DDR-associated parameters in cell lines at baseline. (**A**) Bar charts showing the endogenous/baseline DNA damage in normal and lung cancer cell lines measured by comet assay. (**B**) Redox status expressed by the GSH/GSSG ratio at untreated cell lines. (**C**) Baseline AP-site levels for all cell lines. Error bars represent SD; *** *p* < 0.001. The experiments shown were based on a minimum of three independent repeats.

DDR-related signals were analyzed in two lung cancer cell lines (A549, H1299) and two normal fibroblast cell lines (WS1, 1BR3hT). For all parameters examined, similar results were obtained for the cell lines of each group. First, the endogenous/baseline DNA damage was evaluated using alkaline comet assay, which measures SSBs and/or DSBs. As seen in Figure 1A, the endogenous/baseline DNA damage was found to be significantly higher in lung cancer cell lines than in normal ones (*p* < 0.001), showing accumulation of DNA lesions in malignant cells when there is no known exogenous genotoxic attack. To further investigate the formation of the endogenous/baseline DNA damage, we measured intracellular factors that lead to the formation of SSBs and DSBs, such as redox dysregulation and AP-sites. Interestingly, the GSH/GSSG ratio was found to be decreased, while AP-sites were augmented in cancer cells compared with the normal ones (all *p* < 0.001; Figure 1B,C).

**Figure 2:**
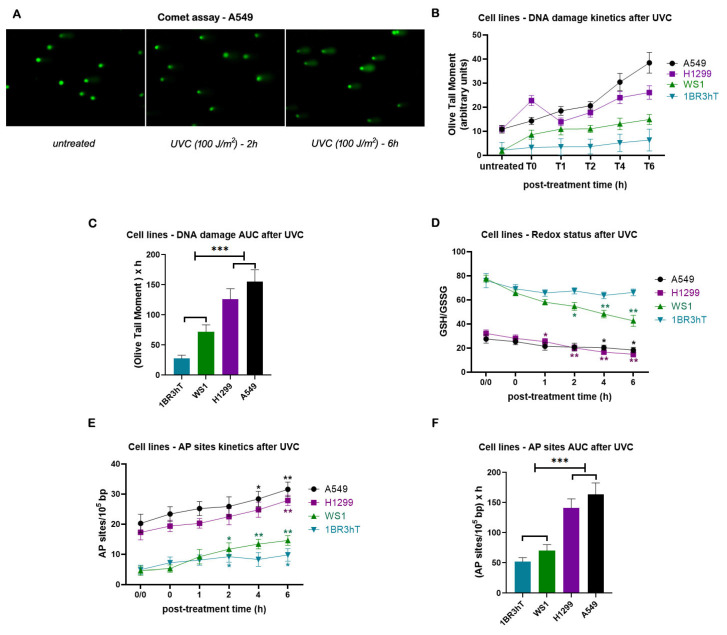
DNA damage response signals in cell lines after UVC irradiation. (**A**) Alkaline comet assay images of A549 lung cancer cell line at baseline and at different time points after UVC irradiation. (**B**) The kinetics of UVC—induced NER—repaired adducts using alkaline comet assay and (**C**) total amounts of DNA damage expressed as AUC for DNA damage during the whole experiment (0–6 h). (**D**) Redox status and (**E**) AP-sites at different time points after UVC irradiation. (**F**) Total amounts of AP-sites expressed as AUC. Error bars represent SD; * *p* < 0.05, ** *p* < 0.01, and *** *p* < 0.001. The experiments shown were based on a minimum of three independent repeats.

Then, the efficiency of NER was evaluated. That is, all cell lines were irradiated with 100 J/m^2^ UVC, which induces cyclobutane pyrimidine dimers (CPDs) and 6–4 photoproducts (6-4PPs), DNA lesions that are repaired by the NER pathway [42], and the DNA damage was measured using an alkaline comet assay (Figure 2A). Significant differences in the efficiencies of NER were found between malignant and normal cell lines. Indeed, lung cancer cell lines showed reduced NER efficiency compared with normal cells (Figure 2B), resulting in higher UVC-induced DNA damage accumulation in malignant cells, expressed as the area under the curve (AUC) for DNA damage during the whole experiment (0–6 h) (*p* < 0.001; Figure 2C). Moreover, in both lung cancer and normal cell lines, we found that UVC irradiation reduced the GSH/GSSG ratio and increased AP-sites (*p* < 0.05; Figure 2D,E). Although kinetic patterns of UVC-induced AP-sites showed no significant differences between lung cancer and normal cells (Figure 2E), augmented accumulation of AP-sites was found in the lung cancer cell lines (*p* < 0.001; Figure 2F), due to increased levels of endogenous/baseline AP-sites in these cells. Together, these data suggest that the increased endogenous/baseline levels of DNA damage found in malignant cells may result, at least partly, from disruption of redox homeostasis and the subsequent formation of AP-sites.

**Figure 3:**
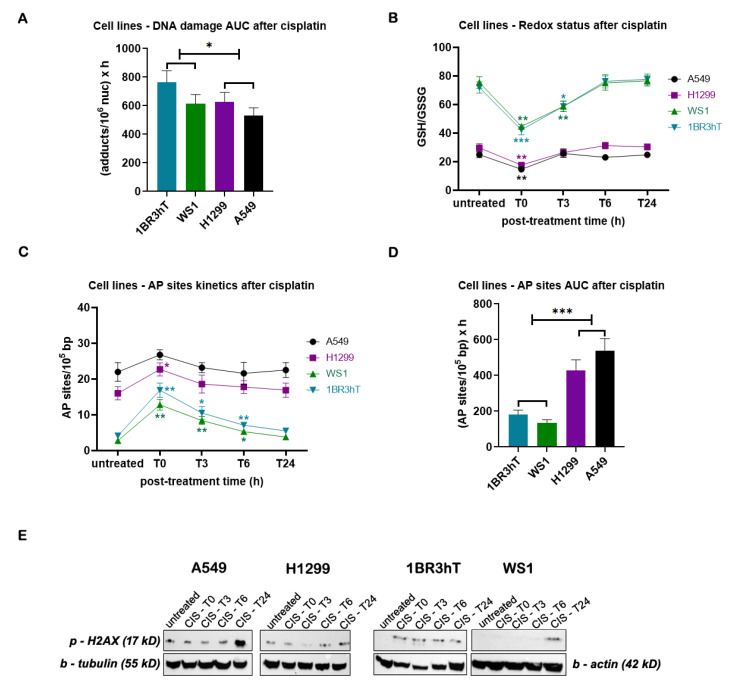
DDR-associated parameters in cell lines following cisplatin treatment. (**A**) The total amounts of cisplatin-induced ICLs expressed as AUC for DNA damage. (**B**) Redox status and (**C**) AP-sites at different time points after cisplatin treatment. (**D**) Total amounts of AP-sites expressed as AUC. (**E**) Western blots showing the amounts of γH2AΧ at different time point after cisplatin treatment. β-tubulin and β-actin were used as loading controls. Error bars represent SD; * *p* < 0.05, ** *p* < 0.01, and *** *p* < 0.001. The experiments shown were based on a minimum of three independent repeats.

To study the efficiency of the ICL repair, cell lines were treated with 25 μg/mL cisplatin for 3 h, and the kinetics of ICL repair was followed for up to 24 h after treatment. Significant differences in the cisplatin-induced ICL burden were found between malignant and normal cell lines, with lung cancer cells showing slightly higher ICL repair capacity (*p* < 0.05; Figure 3A). In addition, in both lung cancer and normal cell lines, cisplatin treatment resulted in a reduction of the GSH/GSSG ratio (Figure 3B) and higher levels of AP-sites (Figure 3C); maximal effect on both factors analyzed was observed at the end of the 3 h cisplatin treatment (time point, T0). Importantly, significant differences were observed in the GSH/GSSG ratio and AP-sites kinetic patterns after cisplatin treatment between lung cancer and normal cells, with the malignant cells returning to baseline levels much faster than normal cells. Moreover, robustly higher total amounts of AP-sites expressed as AUC were found in malignant than in normal cell lines (Figure 3D). Next, the cisplatin-induced phosphorylation of H2AX at the serine residue 139, as a marker of DSBs, was also evaluated. In all cell lines analyzed, maximal levels of γH2AX were observed at the 24 h time point (Figure 3E).

### 3.2. DDR Signals in PBMCs from Patients with Lung Cancer

To test the hypothesis that DDR-associated signals and redox status are implicated in the response to platinum-based chemotherapy, changes in the DDR parameters and the GSH/GSSG ratios were evaluated in PBMCs from 20 healthy controls and 32 patients with lung cancer at baseline (17 responders and 15 non-responders to subsequent platinum-based chemotherapy).

**Figure 4:**
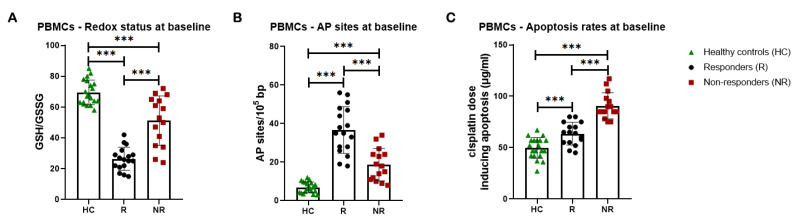
DDR-associated parameters in PBMCs at baseline (**A**) Bar charts showing the endogenous/baseline redox status expressed by the GSH/GSSG ratio at PBMCs from healthy controls (HCs) and patients, responders (Rs), and non-responders (NRs) to subsequent chemotherapy-based treatment. (**B**) Baseline AP-sites levels for PBMCs. (**C**) Apoptosis rates at baseline for PBMCs. Error bars represent SD; *** *p* < 0.001. The experiments shown were based on a minimum of three independent repeats.

Firstly, factors implicated in the formation of DNA damage were evaluated. In line with our previous data [43] and the cell lines’ results, compared with PBMCs from healthy individuals, patients’ cells exhibited significantly lower GSH/GSSG ratios (Figure 4A) and higher burdens of AP-sites (Figure 4B) at baseline (all *p* < 0.001). Interestingly, responders to subsequent chemotherapy were characterized by a significantly lower baseline GSH/GSSG ratio and higher baseline levels of AP-sites compared to non-responders (all *p* < 0.001; Figure 4A,B). In addition, the lowest doses of cisplatin required for the induction of apoptosis at 24 h were significantly higher in PBMCs from patients at baseline compared with healthy controls (all *p* < 0.001), indicating that patients’ PBMCs exhibited significantly decreased apoptosis rates (Figure 4C). In particular, non-responders at baseline exhibited significantly lower apoptotic rates than responders, as their samples required the highest cisplatin dose for apoptosis induction (all *p* < 0.001; Figure 4C).

**Figure 5:**
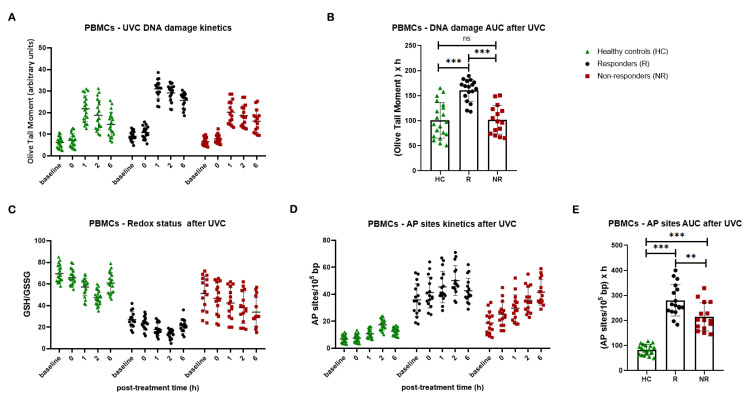
DDR signals in PBMCs after UVC irradiation. (**A**) The kinetics of UVC-induced DNA lesions using alkaline comet assay and (**B**) total amounts of DNA lesions expressed as AUC in PBMCs from healthy controls and patients with lung cancer. (**C**) Redox status and (**D**) AP-sites at different time points after UVC irradiation of PBMCs. (**E**) Total amounts of AP-sites expressed as AUC. Error bars represent SD; ** *p* < 0.01, and *** *p* < 0.001. The experiments shown were based on a minimum of three independent repeats.

Subsequently, NER efficiency was analyzed in PBMCs following irradiation with 5 J/m^2^ UVC. In line with our previous data [43] and the cell lines’ results, significantly lower NER capacity was observed in patients with lung cancer compared with healthy controls, resulting in higher accumulation of NER-repaired lesions in patients’ PBMCs (*p* < 0.01). Intriguingly, patients who responded to subsequent platinum chemotherapy showed significantly lower rates of NER compared with both non-responders and healthy controls, resulting in a significantly higher DNA damage burden in responders’ cells (all *p* < 0.001; Figure 5A,B). Non-responders exhibit similar DNA damage levels to healthy controls, suggesting that impaired NER efficiency might be crucial to chemotherapy response.

In addition, we found that, in a 6 h time frame after UVC irradiation, patients’ samples were characterized by a lower GSH/GSSG ratio and higher UVC-induced AP-sites than healthy controls, with responders showing the lowest GSH/GSSG ratio and the highest levels of AP-sites (all *p* < 0.01; Figure 5C–E).

**Figure 6:**
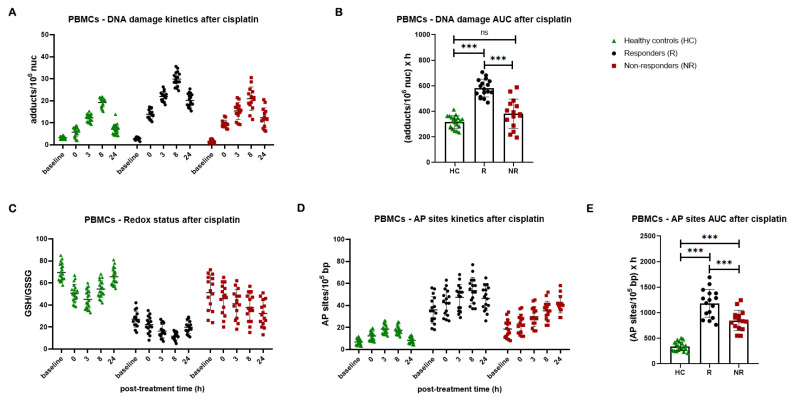
DNA damage response parameters in PBMCs after the ex vivo cisplatin treatment. (**A**) The kinetics of cisplatin-induced ICLs and (**B**) total amounts of ICLs expressed as AUC for DNA damage in PBMCs from healthy controls and patients with lung cancer. (**C**) Redox status and (**D**) AP-sites at baseline and after cisplatin treatment. (**E**) Total amounts of AP-sites expressed as AUC in PBMCs. Error bars represent SD; *** *p* < 0.001. The experiments shown were based on a minimum of three independent repeats.

Next, to investigate the ICL repair efficiency, PBMCs were treated ex vivo with 5 μg/mL cisplatin for 3 h, and the ICL levels were analyzed up to 24 h after treatment. We found that patients with lung cancer showed significantly lower ICL repair capacity than healthy controls, as depicted by higher DNA damage burden after cisplatin treatment (*p* < 0.001). In line with the NER capacity, responders’ cells showed much lower ICL repair capacity than non-responders, resulting in significantly higher accumulation of ICLs in responders’ PBMCs (*p* < 0.001, Figure 6A,B). Once again, non-responders show DNA damage levels equivalent to the ones of healthy controls. In accordance with previous data showing that there is a correlation between the cytotoxicity of bifunctional drugs and the ICL burden (expressed as AUC) [44], we found that the individual levels of cisplatin-induced ICLs (expressed as AUC) correlate with the corresponding apoptosis rates (Figure S1). The GSH/GSSG ratio and AP-sites within 24 h after cisplatin treatment were also evaluated. Significantly lower GSH/GSSG ratio and higher levels of AP-sites were obtained in patients’ samples compared with healthy controls, with responders presenting significantly diminished the GSH/GSSG ratio and augmented levels of AP-sites compared to non-responders (all *p* < 0.001; Figure 6C–E).

## 4. Discussion

The development of drug resistance poses a significant challenge to platinum-based chemotherapy, a crucial treatment regimen employed in the therapeutic management of several malignancies, including lung cancer [45]. Cisplatin’s mechanism of action involves its binding to DNA and the creation of monoadducts, mostly through covalent interactions with guanine’s N7 position. Through a second covalent binding, this monoadduct transforms into a DNA cross-link, which can occur either on the opposite strand (ICL; inter-strand; the most cytotoxic) or on the same DNA strand (intra-strand). Notably, cisplatin does not directly produce double-strand breaks [46]. However, it may still induce double-strand damage when replication forks encounter obstacles to their progression. Indeed, in dividing cells, during the ICL repair, ERCC1-XPF endonuclease makes two incisions on either side of the crosslink in order to release the covalent bond between the two DNA strands. This unhooking event then creates a resection gap, which serves as an appropriate substrate for homologous recombination [47,48,49]. Interestingly, ICL can block the progression of the DNA replication fork, which may lead to the creation of a DNA DSB. In contrast to the direct DSBs that are caused by ionizing radiation, which are typically repaired by non-homologous end joining, these ICL-induced DSBs are repaired by homologous recombination repair [48,49].

Phosphorylation of histone H2AX at position Ser139 (γH2AX) has become a tool to monitor double-strand breaks [50]. Phosphorylation usually happens immediately after the formation of DSBs. However, Olive et al. [51] reported that after exposure to cisplatin, phosphorylation was delayed, reaching maximal levels 6–18 h after drug treatment. They also observed that the time of peak γH2AX levels was cisplatin-dose dependent. This situation was further complicated by the necessity for drug-damaged cells to progress into the S phase. Notably, they found that measurement of γH2AX foci at 24 h post-treatment could serve as a valuable marker of cellular response to cisplatin-induced death. In line with these data, herein, we found that γH2AX protein levels appear shortly after treatment and increase over time, reaching maximal levels within 24 h. Oxidative stress and ROS generation are also linked to cisplatin’s cytotoxic effects [21,24,25,26,27]. Therefore, in this study we tested the hypothesis that redox status and DDR-related signals measured in PBMCs from patients with lung cancer might correlate with therapeutic response to platinum-based therapy.

Firstly, the redox status, expressed as the GSH/GSSG ratio, was assessed. In line with previous studies showing that in cancer cells a number of variables, such as hypoxia, aerobic glycolysis, and oncogene activation, disrupt the redox balance and lead to ROS accumulation, we found that PBMCs from patients with lung cancer showed lower a GSH/GSSG ratio than healthy controls. Prior research has demonstrated a robust association between lung cancer and redox imbalance [52]. In fact, patients with lung cancer had higher levels of oxidative stress biomarkers, including 8-oxodG and malondialdehyde, as well as reduced levels of antioxidative biomarkers, such as red cell superoxide dismutase and glutathione peroxidase activities [53]. Of note, chronic inflammation of the lung tissue is known to be associated with lung cancer [54]. Interestingly, cigarette smoke is known to increase inflammation by raising the number of inflammatory immune cells in the airways and causing the release of proinflammatory cytokines including GMCSF, TNF-α, IL-1, IL-6, and IL-8 [55]. Furthermore, DNA damage can also result in elevated ROS, which can further exacerbate oxidative damage, creating a vicious cycle and raising the burden of DNA damage. Given that the oxidative damage caused by ROS within cells leads to modifications of DNA bases, the greater numbers of AP-sites observed in the patients with lung cancer examined herein may be explained by increased oxidative stress.

Importantly, significant differences in the redox status were found between patients sensitive or resistant to subsequent platinum-based therapy. Indeed, we found that PBMCs from responders are characterized by lower baseline and cisplatin-induced GSH/GSSG ratios compared with patients who are non-responders. Previous studies have shown that the increase in cellular GSH plays a crucial role in cisplatin resistance because of its detoxification effect [56]. That is, before attaching to DNA, many platinum molecules form a Pt(GS)2 conjugate with GSH, which is subsequently removed from the cell. Even though this conjugation may deplete the antioxidant reservoir of the cells and result in oxidative stress, high levels of GSH lower the quantity of reactive cisplatin, thus limiting its anticancer effectiveness [57]. Therefore, it would be expected that an increase in GSH synthesis in cancer cells will cause resistance to platinum-based regimens.

Since the generation of ROS following treatment with many anticancer drugs may augment the treatment efficacy, there is growing interest in combining ROS-inducing agents with chemotherapy [58,59,60]. Indeed, pro-oxidative anticancer drugs, including curcumin and its derivatives, Choline Tetrathiomolybdate (ATN-224), 15-Deoxy-Delta-12,14-prostaglandin J2 (15d-PGJ2), 2-Methoxyoestradiol, and carnosol, are in various stages of research and development [61]. On the other hand, oxidative stress disrupts cellular processes, including the regulation of the cell cycle, apoptosis, and DNA repair mechanisms that are essential for antineoplastic agents to exert their maximum cytotoxicity on cancer cells [62]. This results in the increased lipid peroxidation products, the decrease in blood plasma’s capacity to trap radicals, the reduction of the plasma levels of beta-carotene, vitamin C, and vitamin E, the induction of oxidative DNA damage, as well as the decrease in tissue glutathione levels after chemotherapy [63]. Thus, oxidative stress can also adversely affect normal tissues that undergo rapid proliferation, such as the heart, liver, lungs, kidneys, and gastrointestinal system [64]. Moreover, other adverse events, such as tumor cell adaptation to oxidative stress and cell cycle changes by oxidative stress, decrease the effectiveness of chemotherapy and induce cancer metastasis and recurrence [65]. Interestingly, combination treatment including antioxidants to reduce the side effects of chemotherapy might potentially decrease the efficacy of anticancer agents [66].

Since cisplatin cytotoxicity is mainly due to its ability to cause DNA damage, DNA repair capacity is expected to be one of the most important cisplatin-resistance mechanisms. In this study, we found that patients with lung cancer exhibited decreased NER and ICL repair capacities compared with healthy controls. NER pathway is an important repair mechanism, as it is responsible for the repair of a variety of DNA lesions caused by multiple factors such as UV light, ionizing irradiation, ROS, and chemotherapeutic drugs, including cisplatin [67]. Evidence has suggested that this pathway may be inhibited in lung cells exposed to tobacco smoke [68], with a recent study showing correlations between NER mutations and smoking status in patients with NSCLC [69]. Specifically, an ERCC1 genetic polymorphism was found to be increased in patients who are heavy smokers with NSCLC [69]. ERCC1 is known to be indispensable for the NER pathway, and it has been previously implicated with chemoresistance in lung adenocarcinoma [70]. Moreover, our previous study has shown deregulation of the genes encoding the NER-related molecular components of the heterodimer DDB complex (DDB1 and DDB2) in patients with lung cancer [52]. In line with the NER results, herein we found that PBMCs derived from patients with lung cancer are characterized by decreased ICL repair capacities. In addition, we found reduced apoptosis rates in patients with lung cancer. These results are in accordance with previous data showing that the absence of apoptotic regulation prolongs the life of cancer cells and provides more time for mutations to accumulate, which might enhance invasiveness as the tumor grows, deregulate differentiation pathways, and promote angiogenesis [43]. Corresponding results on most DDR-related parameters were also obtained in cell line experiments, thus further validating the broad applicability of our results. Interestingly, lung cancer cell lines additionally showed higher endogenous/baseline DNA damage (both single- and double-strand breaks) compared to normal fibroblasts, partly due to the elevated levels of oxidative stress that were found in malignant cell lines.

Importantly, significant differences in the NER and ICL repair capacities were observed between lung patients sensitive or resistant to subsequent platinum-based therapy. Indeed, following treatment with UVC or cisplatin, limited lesion accumulation in non-responders’ PBMCs was found, probably emerging from excessive DNA repair activity of both NER and ICL repair mechanisms, resulting in strong resistance to apoptosis in non-responder samples. These results are in line with previous studies in solid tumors, including lung cancer [71,72,73,74], head and neck cancer [75,76,77,78], ovarian cancer [52,79,80], testicular cancer [79], and colorectal cancer [22], as well as in hematologic malignancies [44,81,82,83,84]. These results suggest that it might be possible to predict chemotherapy outcomes by measuring DDR signals in PBMCs derived from patients with cancer.

A limitation of the present study is that we measured DDR-related parameters in the whole PBMC fraction derived from patients with lung cancer. Since PBMCs are not a homogeneous cell population but are composed of various cell types, such as T cells (45–70%), B cells (5–10%), monocytes (10–30%), natural killer cells (NKs; 5–20%), and dendritic cells (0.5–2%) [85], it is important to determine if subpopulations of PBMCs might vary in their response to DNA damage. Therefore, analyzing DDR-associated parameters in the subpopulations of PBMCs from patients with lung cancer with different response rates is an important direction for our future research.

## 5. Conclusions

In order to protect against genotoxic effects, cells have developed a number of genome-protection mechanisms, which are collectively referred to as the DNA damage response network. Interestingly, dysregulation of this system has been linked to the onset and progression of multiple diseases, such as cancer, as well as the response to therapies that cause damage to DNA. In addition, multiple diseases, such as cancer, are caused by disruption of redox homeostasis, which is necessary for human health, with oxidative stress also playing a crucial role in the cytotoxicity of platinum drugs. Therefore, herein we investigated the relationship between the therapeutic benefit of platinum-based regimens, the redox status, and the DDR-related signals of PBMCs derived from patients with lung cancer at baseline. We found that redox status expressed as the GSH/GSSG ratio, the apurinic/apyrimidinic sites, the DNA repair capacity of critical DNA repair mechanisms, namely NER and ICL repair, and the apoptosis rates display significant differences among patients and correlate with the clinical responses to platinum-based therapy. These findings might be exploited as tools to design novel non-invasive predictive biomarkers and might contribute to the identification of patients with lung cancer who are more likely to benefit from this type of therapy.

## Figures and Tables

**Table 1:** Patients and disease characteristics.

Patients (*N = 32*)			
Characteristic	*N*	Years	% of Total
**Sex**			
Male	24	-	75
Female	8	-	25
**Age**			
Median	-	67.5	-
Range	-	49–82	-
**Histology**			
Squamous	10	-	31.3
Non-squamous	17	-	53.1
Small cell	5	-	15.6
**Stage**			
I–II/LD	4	-	12.5
III	7	-	21.9
IV	21	-	65.6
**Smoking**			
Never	3	-	9.3
Current	3	-	9.3
Former	24	-	75
**PD-L1 expression**			
<1%	7	-	21.9
1–50%	7	-	21.9
>50%	7	-	21.9
**Therapy**			
Chemotherapy	10	-	31.3
Chemotherapy–radiotherapy combination	8	-	25
Chemotherapy–immunotherapy combination	12	-	37.5
Chemotherapy–immunotherapy–radiotherapy combination	2	-	6.2
**Cycles of platinum therapy**			
<4	5	-	15.6
4–5	23	-	71.9
6	4	-	12.5
**Response**			
PR	17	-	53.1
SD	5	-	15.6
PD	10	-	31.3

## Data Availability

The data presented in this study are available by specific request to the corresponding author.

## Supplementary Materials

The following supporting information can be downloaded at https://www.mdpi.com/article/10.3390/cancers16244218/s1: Figure S1: Correlation between the cisplatin-induced ICL burden and the apoptosis rates.
