# Supplementary material for: DNA Damage Response Network and Intracellular Redox Status in the Clinical Outcome of Patients with Lung Cancer
Source: Cancers (Basel). 2024 Dec 18;16(24):4218. doi: 10.3390/cancers16244218 (PMC11726754; doi:10.3390/cancers16244218)
Supplement: Supplementary file 1 [file cancers-16-04218-s001.zip › cancers-3362499-supplementary.pdf]

Supplementary Materials

DNA damage response network and intracellular redox status in the clinical outcome of lung cancer patients

Mavroeidi et al.

Table of contents

Figure S1. Correlation between the cisplatin-induced ICL burden and the apoptosis rates.

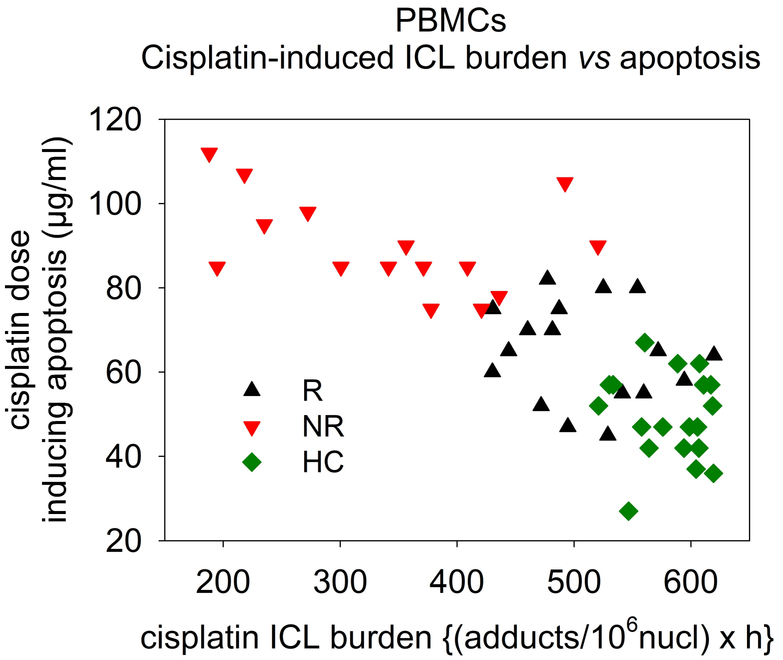

Figure S1. Correlation between the cisplatin-induced ICL burden and the apoptosis rates. The cisplatin-induced ICL levels 0, 3, 6 and 24h following treatment of all lung cancer patients (responders and non-responders) and healthy controls were calculated and the corresponding AUC for ICL during the whole experiment (0-24h) were plotted against the individual apoptosis rates.
